# Why parents refuse childhood vaccination: a qualitative study using online focus groups

**DOI:** 10.1186/1471-2458-13-1183

**Published:** 2013-12-16

**Authors:** Irene A Harmsen, Liesbeth Mollema, Robert AC Ruiter, Theo GW Paulussen, Hester E de Melker, Gerjo Kok

**Affiliations:** 1 National Institute for Public Health and the Environment (RIVM), Centre for Infectious Disease Control, P.O. Box 1, 3720, BA Bilthoven, The Netherlands; 2 Department of Work & Social Psychology, Maastricht University, P.O. Box 616, 6200, MD Maastricht, The Netherlands; 3 TNO Healthy Living (Netherlands Organization for Applied Scientific Research), P.O. Box 2215, 2333 AL Leiden, The Netherlands

**Keywords:** Childhood vaccination, Immunization, On-line focus group, Qualitative study, Decision-making, Beliefs

## Abstract

**Background:**

In high income countries, vaccine-preventable diseases have been greatly reduced through routine vaccination programs. Despite this success, many parents question, and a small proportion even refuse vaccination for their children. As no qualitative studies have explored the factors behind these decisions among Dutch parents, we performed a study using online focus groups.

**Methods:**

In total, eight online focus groups (n = 60) which included Dutch parents with at least one child, aged 0–4 years, for whom they refused all or part of the vaccinations within the National Immunization Program (NIP). A thematic analysis was performed to explore factors that influenced the parents’ decisions to refuse vaccination.

**Results:**

Refusal of vaccination was found to reflect multiple factors including family lifestyle; perceptions about the child’s body and immune system; perceived risks of disease, vaccine efficacy, and side effects; perceived advantages of experiencing the disease; prior negative experience with vaccination; and social environment. The use of online focus groups proved to be an effective qualitative research method providing meaningful data.

**Conclusion:**

Information provided by the NIP turned out to be insufficient for this group of parents. More trust in the NIP and deliberate decisions might result from increased parental understanding of lifestyle and disease susceptibility, the impact of vaccinations on the immune system, and the relative risks of diseases and their vaccines. The public health institute should also inform parents that the NIP is recommended but non-mandatory.

## Background

In recent decades, vaccine-preventable diseases have been greatly reduced through routine vaccination programs in high income countries [1]. In The Netherlands, the National Immunization Program (NIP) is a voluntary program that offers childhood vaccinations free of charge and includes vaccines against twelve infectious diseases (i.e., polio, diphtheria, tetanus, pertussis, rubella, measles, mumps, disease caused by *Haemophilus influenzae* type b, meningococcal C disease, hepatitis B, pneumococcal disease and cervical cancer caused by human papillomavirus (HPV)). Children aged 0–4 years receive the vaccines at child welfare centres (CWC), where they also get free-of-charge health check-ups during consults attended alternately by physicians and nurses on a scheduled basis. Parents can choose between a regular CWC or a CWC based on anthroposophy, a spiritual philosophy founded by Rudolf Steiner [2]. The Dutch Health Council recommends the vaccines included in the NIP, and the National Institute of Public Health and the Environment (RIVM) manages the program implementations of the NIP and provides parents and health care workers with information about vaccinations. Parents of infants receive some oral information about the NIP when a nurse of the CWC visits the parents at their home in the first week after birth of their infant. After that (when the child is 4–6 weeks old), parents receive a brochure with information about vaccines, (vaccine-preventable) diseases, vaccination schedules, and side effects.

Overall, vaccination coverage in The Netherlands is 95% (except for HPV) [3]. Despite the success of the NIP, many parents appeared to become more critical about childhood vaccination in the last few years, at least as far as HPV-vaccination is concerned. In The Netherlands, there was a wide debate in the national press about the 2009 introduction of vaccination for HPV for 12 year old girls, resulting in mixed messages and confused feelings in the population [4]. The expected HPV vaccination coverage of 70% turned out to be about 50% [5]. Also, at the end of 2009, during the H1N1 influenza pandemic, Dutch parents criticized the quality of information about the risks and benefits of the influenza vaccination, which was provided by the national health authorities [6]. A well-known group who refuse vaccination are conservative Protestants living in what is called the Bible Belt region, which stretches from the southwest to the northeast of the country. Such refusals have been influenced by tradition or predominantly religious arguments [7]. Parents who refuse vaccinations might also be influenced by other factors.

Refusal of childhood vaccination may be influenced by concerns about vaccine components, low perceived likelihood and severity of the infectious diseases, and a trusting relationship with a natural healer or another respected person who doubts vaccination safety and effectiveness [8,9]. Hilton et al. [10] showed that some parents fear an overload of the immune system caused by combination vaccines. Additionally, the perception that vaccination is more risky than non-immunization [11] and issues of harm, distrust and access might play a role in refusing childhood vaccination [12]. According to Sporton et al. [13], parents who refused vaccination made a well-considered decision based on an assessment of the benefits and the risks of vaccination, the child’s susceptibility to the potential disease, and the acceptance of responsibility for that decision.

The aim of this study is to attain more insight into these factors in order to design public information and interventions that will help parents make decisions that best serve their children and the wider community. We performed internet-based focus groups with parents who had refused all or part of the NIP recommendations for children 0–4 years old.

## Methods

The focus group discussions were conducted online because the diverse population was difficult to reach and lived throughout The Netherlands, making face-to-face focus groups infeasible. Online focus groups are used more and more [14], in part because participants can choose their own time to answer questions. Moreover, costs and time can be saved through the automatic and accurate storing of discussion data [15]. The focus group method in general is effective for exploring people’s opinions and experiences [15]. The group process can help individuals to clarify their views that might not emerge from a one-on-one interview.

### Study participants

Study participants were randomly selected from Praeventis, the vaccination database in The Netherlands. Participants were selected based on the vaccination status of their children (0–4 years old). Postal codes were used to exclude residents of the Bible Belt, whose reasons for refusing vaccinations have been explored by others [7]. We invited 250 parents with partially vaccinated children (PV parents) and 250 parents with children not vaccinated at all (NV parents). We defined children (aged 0–4 years) as partially vaccinated when they missed one or more NIP vaccinations, and as not vaccinated when they missed all vaccinations in the program.

### Procedure

Parents received a letter containing information about the study and a reply form to complete and return if they wanted to participate. Those opting to participate received an e-mail with information about the use of the online focus groups and a personal log-in name and password, by which they could anonymously access the online forum. When participants responded to each topic discussed by the online group they received a gift voucher of €30 as an incentive. Of the researchers, only the moderator and assistant had access to the forum, for collecting the data. Anonymity of statements in the transcripts and in the final report was ensured, as was confidentially of the data. Because data collection was through the Internet, participants gave informed consent by clicking a button after having read all relevant information. The study was approved by Maastricht University’s Ethics Committee of Psychology.

### Study setting

The focus groups were based on a semi-structured protocol with open-ended questions and minimal control, allowing participants to discuss all aspects of each posting. The list of topics was developed beforehand, in consultation with all the authors, and subsequently tested with other colleagues who had young children. Each of the online focus groups was conducted over 5 days during one week. The focus groups ran during November and December 2011. The forum was accessible only to parents who had responded to the invitation letter and received a log-in name and password. Each week, Monday through Friday, the moderator posted a new topic at the forum daily, and the group participants were alerted by e-mail. All postings remained open for response throughout the week. The focus groups were asynchronic, which means that participants were free to log into the forum discussions at any time to read all postings and respond within one week. The moderator regularly checked the forum and, when necessary, asked additional questions to clarify comments of participants. The content and format of postings were identical for all focus groups.

The forum for each group started with an introduction and with questions for participants about their family composition, the CWC that they visited, and perceived positive and negative aspects of the NIP. On the second day, parents were asked which factors influenced their decision to refuse any or all vaccinations. On the third day, they were asked about their need for NIP information. The fourth day focused on their perceptions about new vaccines within the NIP. The fifth day was used to end the discussion, with an evaluation of the focus group by the participants. After conducting 8 sets of focus groups, analysis indicated that data saturation had been reached, making the inclusion of more respondents unnecessary.

### Analysis

The data was analyzed based on a thematic analysis [16] performed to explore factors that influenced parents’ decision to refuse vaccination. The main themes of the data were based on the topics and questions posted at the online forum. An inductive process was used to code and analyze the data for the sub-themes from these main themes. The data was analyzed and coded by the moderator. An independent researcher analyzed a sample of the data; afterwards the initial coding was compared, reviewed, discussed, and refined until consensus could be achieved, which led to a more representative coding scheme and criteria. Using software program NVivo 9 (QSR International), separate analyses were conducted for PV parents and NV parents.

## Results

### Participants

In total, we held 8 one-week online focus groups with all the parents who responded to the invitation (n = 60) and who had refused all or part of NIP vaccinations on non-religious grounds. Of the 8 groups, 5 included parents who completely refused vaccinations (n = 39, 7–9 parents each), and 3 included parents who partially refused vaccinations (n = 21, 7 parents each).

Five parents had one child; most parents had two (n = 34) or three children (n = 14); 6 parents had four children, and one parent had five children. Most parents visited a regular CWC (NV = 25, PV = 19), some parents visited an anthroposophical CWC (NV = 10, PV = 1), and some parents used no CWC at all (NV = 4, PV = 1). Because of the anonymity of the participants, no other demographic variables (like gender) were available.

The four main themes (i.e., topics at the online forum) were divided into sub-themes and are summarized below with relevant quotes of the participants. Despite separate analyses, the findings on parents who partially and completely refused vaccination are described together, because they were very similar. The few differences between these two sub-groups are described at the end of the results section.

### Positive and negative aspects of the NIP

Regarding theme one, PV and NV participants were asked to mention some positive and negative aspects of the NIP in general. Participants agreed that a positive aspect of the NIP is that it is well organized: *“It is a well-organised 'machine’” (NV).* Another positive is that vaccines are freely available. Participants who realized that the NIP is non-mandatory felt positive about this, too: *“A positive aspect is that you have access to vaccines in The Netherlands and, as a parent, you have a free choice” (PV).*

Some participants mentioned that there were too many vaccines and that vaccination in the NIP started too early: *“A negative is that more and more vaccines are added” (PV)*. Another participant said: *“I find it unfortunate that the RIVM vaccinates at a very young age when the immune system is hardly built” (NV*). Another negative aspect that participants agreed upon was that they felt vaccination was mandatory, although it is not: *“You get the feeling that you MUST do it. When you do not vaccinate you receive a reminder to vaccinate by post. You feel almost guilty if you do not participate” (PV).*

### Determinants of vaccine refusal

Theme two focused on parental decision-making. Various factors influenced the choice of parents to refuse vaccination partially or completely. These were related to lifestyle and parental perceptions about the body and the immune system of the child, risk perception of diseases and vaccination side effects, perceived vaccine effectiveness, the potential advantages of experiencing the disease, negative experiences with vaccination, and social environment.

#### Lifestyle

Lifestyle of the participants appeared an important determinant for refusing vaccination. Participants mentioned that their healthy lifestyle promotes their children’s health, and therefore the risk of getting an infectious disease is reduced. Some participants focused only on nutrition: *“We rely on our 'preventive’ eating habits and lifestyle. Especially good nutrition ensures that you do not get ill” (NV).* Other participants focused on other aspects of a healthy lifestyle, such as giving children a peaceful basis for life: *“All my choices are currently aimed to give my children a peaceful basis for life: choose to breastfeed (about 1.5-2 years), raise children in a small-scale home, part-time work, first half-year no childcare, minimize shopping/travelling with young children. All kinds of things that do not overcharge the immune system” (NV).*

#### Immune system

Most participants also mentioned that they believed that the immune system of the child was not yet adequately developed to receive vaccinations: *“Administering many different viruses/bacteria at the same time seems to me a huge attack on the immune system of someone” (NV).* Another participant said: *“A baby’s immune system has built up thanks to the mother, and it is not desirable in my eyes to give the child all kinds of substances that can disrupt the whole immune system” (PV).*

#### Risk perception disease

The risk perception of the disease is low, because some participants seemed to think that their children were not likely to contract infectious diseases and that infections were not likely to be transmitted to their child: *“I also assumed, based on the fact that both children did not come that much in contact with other children at a very young age, that the risks [of getting the disease] were less” (PV).* Furthermore, some participants mentioned that vaccine-preventable diseases are not that severe and can be easily treated: *“Most of them [the diseases] are not life threatening and, with support of the family paediatrician or homeopathic doctor, they are easy to treat” (NV).*

#### Risk perception of vaccine side effects

Participants who perceived little risk of the disease accordingly believed that the likelihood of negative consequences of vaccination is higher and that these consequences are more severe than getting the disease: *“There are many unpleasant side effects and diseases that are due to the vaccinations, and this is always dismissed as untrue” (PV).* One participant said: *“We also have serious doubts about the consequences of vaccinations. […] We also see a link between vaccinations and some behavioural problems” (NV).* Other participants doubted certain components of the vaccines: *“They also get many germs at once, I consider this mechanism unproven” (PV).* Another participant doubted about the negative consequences of the adjuvants in vaccines: *“There are adjuvants in vaccines that are poisonous, such as mercury and aluminium, and you really do not want that in your body, even in small quantities” (NV).*

#### Perceived efficacy vaccine

Participants were also worried whether or not vaccine efficacy is adequate and if vaccines would lead to protection: *“Some diseases are obsolete and disease agents mutate, so the protection is not always 100%. Some vaccines work only temporarily, while the side effects may be permanent (i.e., allergies, chronic colds, autism etc.). Even though children were vaccinated, there are still epidemics (such as mumps, whooping cough)” (PV).* Another participant said: *“I refused vaccination against pertussis, because the effect of pertussis vaccination does not seem to be large. More and more people get pertussis, despite new vaccines and the fact that children get vaccinated at a younger age” (NV).*

#### Perceived advantages of having a disease

Some participants believed that attracting a vaccine-preventable disease was something positive for their child(ren). These participants cited the advantage of life-long immunity: *“Let the body itself go through the disease. This is good for building up the resistance by the body itself. Diseases often give life-long immunity, while vaccines often protect for only 15 years” (PV).* Some participants believed a child would develop physically and/or mentally after getting a disease: *“You could say that the experience of a disease has a particular function; it makes a certain physical and/or mental development possible” (NV).*

#### Negative experience with vaccination

A negative experience with childhood vaccination influenced the decision making of participants. Some participants were influenced by a negative story in the media: *“Two years ago there was the case in which something went wrong with vaccinations for young children. Shortly after that, we refused a vaccination” (PV).* Some have had a negative experience in their own environment: *“Death in the family within 24 hours after vaccination…made me gain more in-depth knowledge. Together we made the choice not to vaccinate” (NV).* Others cited a very personal negative experience: *“Our oldest daughter (10 years) got epilepsy after vaccination. She got attacks for forty-five minutes. It was not clear to us that it was because of the vaccinations until she got such a heavy attack after the MMR vaccination that she ended up in intensive care. It’s unbelievable, but doctors deny any form of adverse reactions following vaccination” (NV).*

#### Social environment

There were mixed findings as to whether people in the social environment influenced the parental choice to refuse vaccination. Some participants said their environment had not influenced their choice at all, whereas others said they were influenced by their friends or family members: *“In my environment I had one friend who also looked critically at vaccinations. Partly because of that, I gained more in-depth knowledge” (PV).* Another participant said: *“I had a conversation with my mother and sister about whether to vaccinate or not. My sister did not adhere to the vaccination schedule; she vaccinated her children later than recommended” (NV)*. Other participants indicated that no one in their environment influenced them: *“No people in our environment influenced our decision. We didn’t know people who were critical towards vaccination” (NV).*

Interestingly, some participants said that they did not talk about their choice to refuse vaccination with others in their environment, because they expected negative reactions: *“In my environment, I sometimes have to defend why we do not follow 'the norm’ [to vaccinate]” (PV).* Another participant said*: “We are the only ones who did not vaccinate! Our choice has often led to discussions, and more than once people showed that they thought we were crazy” (NV).*

### Need for information

Theme three focused on the informational needs of participants. Many mentioned that they did not receive enough information from the RIVM about childhood vaccination: *“Negative to the NIP, I think, is that parents get absolutely no information about the vaccines. A box of paracetamol has a leaflet with a big piece of text, but about vaccinations we are only told that the puncture site may be painful, or that the child can get some fever” (PV).* Participants indicated they would like to get more information about their freedom of choice: *“I miss strong objective information about the background and choice options that you have as a parent, like vaccinating later…or choosing some vaccinations but not others” (PV).* Specific information about the possible negative consequences of vaccines, like side effects, is also needed: *“I also think that parents are not fully informed about the side effects and ingredients of vaccines by the RIVM.” (PV)* Another participant stated: *“I would like to have open and honest information, whereby the disadvantages and risks of vaccination are discussed so parents could make a well-considered decision” (NV).*

Because participants’ information need was not fulfilled, they started to seek information by themselves. Some said that it was hard for them to find the right information and to make a choice to vaccinate or not, based on all the positive and negative information they found. One said: *“Although I am trained to read and evaluate research, I had great difficulty to find my way in all the information” (NV)*. Another said: *“We searched for all kinds of information, and the problem is: there is too much and you do not know how to filter. What is an opinion, what is a fact? Who is trustworthy, who is not?” (NV).*

### New vaccines in the NIP

Theme four focused on possible new vaccines being added to the NIP in the future. Participants had mixed feelings whether they would accept new vaccines or not. Some said that they would refuse all new vaccines in advance, because there are already enough vaccines in the NIP: *“Even more vaccinations? My goodness, I think it is already too much! Let nature take its own course, please” (PV).* Other participants said they would weigh the pros and cons of each new vaccine and make a deliberate choice: *“Facing new vaccines, we think the same as compared with existing vaccines: how is the vaccine tested? What is exactly in it? What would be the side effects, etcetera. We are not fundamentally against it” (PV).*

### Differences between NV and PV parents

Participants who partially or completely refused vaccination reported many similarities in the way they think and make decisions about vaccination. However, there were still some differences between the two groups. For example, participants who completely refused vaccination reported having positive experiences with not vaccinating their child(ren). They mentioned that compared to children who were vaccinated, their unvaccinated children were less often sick: *“It is our experience that our child, compared with vaccinated children at his age within our environment, is less sick, and when he is sick he recovers more quickly” (NV).* The participants who completely refused vaccination also discussed herd immunity, saying it was not a reason they refused vaccination. They did not depend on it to protect their unvaccinated child. Indeed, some regretted the presence of herd immunity because it reduces the chance that their child will get the disease and thereby develop natural immunity against the disease: *“It is absolutely not true that our children have not been vaccinated because others do. I rather hope that my children get certain childhood illnesses at a young age than (because of the high vaccination coverage) getting the disease when they are older” (NV).* These participants also mentioned that they trusted the health care in The Netherlands and believed that when their child gets sick, the quality of health care is good enough to take care of their child: *“We rely on the various methods of treatment, both conventional and alternative, when we face serious diseases” (NV).*

Among PV participants, we found that some had not thought beforehand about refusing a certain vaccination. Some refused or postponed vaccination simply because their child was sick at the time, and therefore was not able to receive the vaccine: *“I followed my feelings and did not vaccinate my child especially when I suspected that something was troubling, like a cold or some other inconvenience” (PV).* Another participant said: “*The main reason [to not vaccinate] was that my daughter struggled with her health, and that I first wanted that she would be healthy before she got vaccinated” (PV).*

## Discussion

This study explored what factors are important in refusal of childhood vaccination by parents. Like Sporton et al. [13], we found that most refusal of vaccination is based on deliberate decision-making of parents. Our results show that this decision is based on multiple factors, such as the lifestyle of parents, perceptions about the body and the immune system of the child, risk perception of diseases and vaccination side effects, perceived vaccine effectiveness, perceived advantages of experiencing the disease, negative experience with vaccination, and parents’ social environment. In addition, this study shows that the use of online focus groups is an effective qualitative research method resulting in meaningful data.

An important determinant of refusing vaccination is the lifestyle of parents. Some of our participants stated that living a healthy life decreases the risk of getting an infectious disease. This determinant was also mentioned by Meszaros et al. [17]. This indicates that not only perceptions and beliefs about childhood vaccination are an import factor in parents’ decision to refuse vaccination, but also that the general lifestyle of the parents might play a role.

Another determinant, which has also been reported by other studies [18-20] is risk perception. A 2007 meta-analysis of studies linking risk perception and vaccination by Brewer et al. [21] points to risk perception as an important factor in health behaviour. Our study shows that parents who refuse vaccination believe that the side effects of vaccines could be severe, that vaccine-preventable diseases are not that severe, and that their child is not very susceptible. These beliefs might reflect the fact that vaccine-preventable diseases have been reduced to the point that their risks seem less important than vaccination risks [22]. It therefore seems important that public health institutes keep communicating about the severity and susceptibility of vaccine-preventable diseases.

Besides the perceived risk of disease versus vaccination, our findings as well as those of Hilton et al. [10] suggest that parents fear the immune system in infancy is not adequately developed for a good response to vaccination. They apparently have not received enough information about the influence of vaccines on the immune system of their child, and their resulting doubts cause them to refuse vaccination.

Benin et al. [9] showed that parents who refused vaccination reported a trusting relationship with a natural healer or some other respected person having doubts about vaccination. Our study shows similar results, in that a proportion of the parents visited an anthroposophical CWC. Besides this, some parents mentioned that experiencing a disease is positive, leading to certain physical and/or mental development. This perception seems consistent with the anthroposophical lifestyle and view about vaccination [2]. The vaccination coverage among anthroposophists in The Netherlands is somewhat lower compared to the rest of the population [23], and a study of Harmsen et al. [24] showed that parents who visited an anthroposophical CWC mostly refused the Mumps, Measles, and Rubella (MMR) vaccination because they perceive these diseases as essential for the physical and mental development of their child. These findings might indicate that parents with an anthroposophical lifestyle and/or parents who visit an anthroposophical CWC might be more critical towards childhood vaccination. However, the influence of anthroposophical CWCs on parents’ decision making is so far not clear and therefore more research is needed.

Interestingly, this study showed mixed results about the influence of the social environment. As found previously [25], sometimes parents feel supported in vaccination refusal by their family and friends, with whom they discuss the issue. Others discuss it with no one, in part due to fear of negative responses from their community. Brown et al. [26] mentioned that parents felt that their decision to vaccinate or not would be judged by people around them.

Mills et al. [12] and Brown et al. [26] showed in their studies that forgetting to make an appointment or to schedule an appointment were also factors that influenced a lower vaccination coverage. This factor was not found in this focus group study, future quantitative research is needed to explore this further.

Other studies have shown that parents need more information about childhood vaccination [27-29]. Our study results showed that this is true also for Dutch parents. They would particularly like more information about the side effects of the vaccines, the components of the vaccines and more assurance that the NIP is non-mandatory.

Parents in this study indicated that when they start searching for information, it is hard to find reliable information and to make a choice from all the information they find. RIVM should therefore supply more information about childhood vaccination and also list reliable sources of additional information. In addition to official websites, social media should also be listed because of the growing proportion of online communicators, including vocal and active anti-vaccination groups [30]. Along with the risks of non-vaccination, the official information should address the risks of vaccination. Official language should be moderate, avoiding extreme formulations, because a strong assertion that there is no risk in vaccination can paradoxically lead some people to suspect or perceive a higher risk [31].

Our study has both strengths and limitations. The primary strength is its use of online focus group discussions. At our online forum, parents were anonymous and therefore free to say whatever they wanted. In addition, parents could log in and respond whenever they had time, which might have resulted in a high response to every posting. Besides these strengths of the online focus group, a limitation might be that parents responded less to other parents’ comments compared to face-to-face focus groups, which might have resulted in less discussion. Although qualitative studies do not seek to achieve representativeness through randomization, our study is limited by its lack of demographic information. Such information would have made findings more representative with regard to, for example, gender, educational attainment, and age. Another limitation is a possible response bias, as parents who are more negative about childhood vaccination might have been more willing to participate. Unfortunately, we have no access to information about the background of parents’ non-response to our invitation. While this qualitative study provides useful insight in the factors that influence decision-making about vaccination of parents who refused vaccination, quantitative confirmation of the findings is recommended among a large population of parents to get insight in which determinants are most important.

## Conclusion

This study provides an in-depth insight into the perception of parents who took the deliberate decision to refuse all or part of the free vaccinations in the Dutch NIP. Information currently provided by the RIVM turned out to be insufficient for this group of parents. They are in need of verifiable knowledge about the effects of vaccination on the development of a child’s immune system; how much a healthy lifestyle can, by itself, protect children from vaccine-preventable disease; and what are the real risks, consequences and complications of such disease. At the same time, the information must increase trust in the NIP by providing more detail about vaccine side effects and more assurance that the NIP is not mandatory. Access to additional sources of reliable information should be provided. Listening to critical parents is useful for developing communication strategies that suits their concerns and reduce their feelings of ambivalence in decision making about childhood vaccinations. Further study is needed on how such information could best reach the parents who need it.

## Competing interests

The authors declare that they have no competing interests.

## Authors’ contributions

IH develop the study design, performed the data collection, data analysis and wrote the manuscript. LM, HdM, RR, TP and GK discussed the study and focus group design. All authors contributed to the draft of the final manuscript; their remarks were discussed and processed into the final version that was finally approved by all authors.

## Pre-publication history

The pre-publication history for this paper can be accessed here:

http://www.biomedcentral.com/1471-2458/13/1183/prepub
